# Integrating Physiology, Cytology, and Transcriptome to Reveal the Leaf Variegation Mechanism in *Phalaenopsis* Chia E Yenlin Variegata Leaves

**DOI:** 10.3390/biom14080963

**Published:** 2024-08-07

**Authors:** Ji Li, Jianqiang Wen, Kunlin Wu, Lin Li, Lin Fang, Songjun Zeng

**Affiliations:** 1 Key Laboratory of South China Agricultural Plant Molecular Analysis and Gene Improvement, South China Botanical Garden, Chinese Academy of Sciences, Guangzhou 510650, China; liji17@scbg.ac.cn (J.L.); wenjq@scbg.ac.cn (J.W.); wkl8@scib.ac.cn (K.W.); lilin@scbg.ac.cn (L.L.); 2 University of Chinese Academy of Sciences, Beijing 100049, China; 3 Guangdong Provincial Key Laboratory of Applied Botany, South China Botanical Garden, Chinese Academy of Sciences, Guangzhou 510650, China

**Keywords:** leaf color, chlorophyll biosynthesis, RNA-Seq, transcription factors, plant hormones

## Abstract

*Phalaenopsis* orchids, with their unique appearance and extended flowering period, are among the most commercially valuable Orchidaceae worldwide. Particularly, the variegation in leaf color of *Phalaenopsis* significantly enhances the ornamental and economic value and knowledge of the molecular mechanism of leaf-color variegation in *Phalaenopsis* is lacking. In this study, an integrative analysis of the physiology, cytology, and transcriptome profiles was performed on *Phalaenopsis* Chia E Yenlin Variegata leaves between the green region (GR) and yellow region (YR) within the same leaf. The total chlorophyll and carotenoid contents in the YR exhibited a marked decrease of 72.18% and 90.21%, respectively, relative to the GR. Examination of the ultrastructure showed that the chloroplasts of the YR were fewer and smaller and exhibited indistinct stromal lamellae, ruptured thylakoids, and irregularly arranged plastoglobuli. The transcriptome sequencing between the GR and YR led to a total of 3793 differentially expressed genes, consisting of 1769 upregulated genes and 2024 downregulated genes. Among these, the chlorophyll-biosynthesis-related genes *HEMA*, *CHLH*, *CRD*, and *CAO* showed downregulation, while the chlorophyll-degradation-related gene *SGR* had an upregulated expression in the YR. Plant-hormone-related genes and transcription factors MYBs (37), NACs (21), ERFs (20), bHLH (13), and GLK (2), with a significant difference, were also analyzed. Furthermore, qRT-PCR experiments validated the above results. The present work establishes a genetic foundation for future studies of leaf-pigment mutations and may help to improve the economic and breeding values of *Phalaenopsis*.

## 1. Introduction

Leaf color in evergreen plants is typically associated with the content and relative ratios of photosynthetic pigments including chlorophyll and carotenoids. Among them, chlorophyll serves as the principal photosynthetic pigment and plays a pivotal role in light harvesting in photosynthetic antenna systems and electron transport in reaction centers [1]. Research on the occurrence, genetic factors, and underlying mechanisms of leaf-color mutants in model plants has been conducted, such as in *Arabidopsis thaliana* [2,3], maize [4], rice [5], and burley tobacco [6]. Furthermore, the study of leaf-color mutations in orchids has garnered increasing attention in recent research. In *Cymbidium sinense*, the RNA-Seq of the mutant leaves revealed that changes in leaf color may be due to excessive degradation of chlorophyll rather than insufficient biosynthesis [7]. Comparative analyses at the phenotypic, physiochemical, cytological, and transcriptomic levels between yellow mutant and normal plants in *Cymbidium* ‘Sakura’ revealed that chlorophyll degradation may contribute to the yellowing leaves [8]. In our previous study, low chlorophyll content and an aberrant chloroplast ultrastructure were observed in the *Paphiopedilum* leaves of the yellow mutant compared with the green plant [9].

To date, a significant number of mutated genes have been confirmed to be involved in leaf variegation, most of which were found to participate in chloroplast biogenesis and the biosynthesis of photosynthetic pigments [10]. For example, previous research in *Arabidopsis* has shown that mutations in FtsH2 or FtsH5 can lead to leaf yellowing, while double mutants of FtsH2 and FtsH8 exhibited an albino phenotype [11,12]. Rice yellow-leaf mutants with a loss of *YS83* gene function exhibited reduced photosynthetic pigment content and delayed chloroplast development during the seedling stage, yet their seed-setting rate and thousand-grain weight were not significantly affected [13]. At the level of transcriptional regulation, the NAC, ERF, bHLH, and MYB transcription-factor families were pivotal in mediating the transcriptional modulation of genes implicated in the biosynthesis or degradation of chlorophyll [14,15,16,17]. Moreover, several plant hormones such as cytokinins, auxins, gibberellins, abscisic acid, jasmonic acid, ethylene, and salicylic acid have also been reported to control chloroplast development, leaf senescence, and leaf yellowing. [18,19,20].

*Phalaenopsis* is an epiphytic monopodial plant in the Orchidaceae family with a fantastic appearance and prolonged flowering period. It is the most commercially developed orchid plant worldwide and has high ornamental and economic value [21,22]. Currently, research on *Phalaenopsis* mainly focuses on functional genes [23,24], in vitro tissue culture [25,26], breeding [27,28], and floral characteristics [29,30], but there has been little research on leaf-color mutation. Leaf color serves as a crucial parameter for assessing the aesthetic attributes of orchid foliage, and orchid cultivars with variable leaf color are limited, resulting in elevated market prices for these variants [8,31]. Therefore, determining the molecular mechanisms responsible for variegation in leaf coloration and expediting the breeding of *Phalaenopsis* with novel leaf traits have emerged as significant areas of research interest [32,33].

In the present study, we integrated cytological, physiological, and transcriptomic analyses to elucidate the mechanisms underlying leaf variegation in the *Phalaenopsis* mutant ‘Chia E Yenlin Variegata’ (a periclinal chimera). We selected the yellow and green regions from the same leaves and analyzed their photosynthetic pigment content, chloroplast ultrastructure, and genes implicated in leaf-color variegation. By analyzing the transcriptome data, we evaluated the gene expression patterns associated with chlorophyll and carotenoid metabolism. Additionally, the potential regulator genes and related pathways were identified. This study provides a critical genetic resource to elucidate the molecular mechanisms underlying leaf-color variegation and facilitate the breeding of new *Phalaenopsis* varieties with enhanced horticultural value.

## 2. Materials and Methods

### 2.1. Plant Materials

Three-year-old plants of the *Phalaenopsis* cultivar ‘Chia E Yenlin Variegata’ were planted in the greenhouse of the South China Botanical Garden, Chinese Academy of Sciences (Guangzhou, China). The plants were exposed to natural light under a 60% shade cloth, and the temperature and relative humidity were maintained within the ranges of 15 to 34 °C and 75–99%, respectively. Mature leaf samples of the green region (GR) and yellow region (YR) (all leaf layers) were collected in April 2022. Samples were immediately flash frozen in liquid nitrogen and then stored at −80 °C in the laboratory. Three biological replicates were performed for all experiments.

### 2.2. Determination of Chlorophyll and Carotenoid Contents

Approximately 0.1 g of fresh leaf tissue from the GR and YR was ground into powder by a MM400 shaker mill (Retsch technology, haan, Germany) in liquid nitrogen and then underwent extraction using 80% acetone at 4 °C overnight. The absorbance readings at 646.8 nm, 663.2 nm, and 470 nm were measured using a microplate reader (Tecan Infinite, Männedorf, Switzerland). The chlorophyll and carotenoid concentrations were determined following the method described by Lichtenthaler [34].

### 2.3. Ultrastructure Observations

The chloroplast ultrastructure was observed following the protocol previously described [9]. Briefly, mature leaf samples from the GR and YR of ‘Chia E Yenlin Variegata’ were cut into 1.0 × 2.0 mm pieces and fixed in 0.1 M PBS (sodium phosphate buffer, pH = 7.2) containing 2.5% glutaraldehyde and 2% paraformaldehyde. After fixation, the samples were rinsed six times with 0.1 M PBS, then postfixed in 1% osmium tetroxide for 4 h and rinsed once more with 0.1 M PBS. The plant tissue fragments were then embedded in epoxy resin Epon-812 after the samples were dehydrated. Ultrathin sections (80 nm) were prepared using a UC7 ultramicrotome (Leica Microsystems, Wetzlar, Germany) and stained with 4% uranyl acetate and 2% lead citrate. The sections were examined for leaf ultrastructure using a transmission electron microscope (JEOL JEM-1010, Tokyo, Japan) at a voltage of 100 kV.

### 2.4. RNA-Seq and Data Analysis

Briefly, 0.1 g of GR and YR tissues was used to isolate total RNA with an RNAprep Pure Plant Kit (TIANGEN BIOTECH, Beijing, China). The integrity, concentration, and purity of the total RNA were evaluated using an Agilent 2100 bioanalyzer (Agilent Technologies, California, USA) and ND-2000 NanoDrop (Thermo Scientific, Wilmington, DE, USA), respectively. In total, 1 μg of high-quality RNA sample was used to construct the RNA-Seq libraries using Illumina^®^ Stranded mRNAPrep (San Diego, CA, USA). The sequencing of cDNA libraries was completed at Shanghai Majorbio Bio-pharm Biotechnology Co., Ltd. (Shanghai, China) on the Illumina HiSeq™ 6000 sequencing platform (Illumina, San Diego, CA, USA). The clean reads were obtained after the sequencing adapters, low-quality reads, and primer sequences were removed from raw reads. The clean reads were then aligned to a *Phalaenopsis equestris* reference genome [35] (https://www.ncbi.nlm.nih.gov/genome/11403) (accessed on 1 August 2024) using Hisat2 (v2.1.0) [36]. The assembled unigenes underwent functional annotation through comparison with the public databases, namely, NCBI non-redundant protein (Nr), Swiss-Prot, Kyoto Encyclopedia of Genes and Genomes (KEGG), Gene Ontology (GO), Protein family (Pfam), and Clusters of Orthologous Groups (COG).

### 2.5. Differentially Expressed Gene (DEG) Analysis

To identify DEGs (differentially expressed genes) between GR and YR tissues, the abundances of the annotated genes were calculated and normalized by the transcripts per million reads (TPM) method using RSEM software (v1.3.3). Differentially expressed genes (DEGs) between the GR and YR samples were identified by comparing their TPM values using DESeq2 (v1.24.0) [37]. Genes with *p*-adjust < 0.05 and log2 (fold change) ≥ 1 were considered to be significantly differently expressed genes and subjected to an enrichment analysis of KEGG pathways.

### 2.6. Verification of Gene Expression Profiling Using RT-qPCR

The total RNA used in transcriptome sequencing was utilized for cDNA synthesis with a TransScript^®^ One-Step gDNA Removal cDNA Synthesis SuperMix Kit (Transgen, Beijing, China). qRT-PCR was performed on a LightCycler 480 II system (Roche, Mannheim, Germany) using PerfectStart Green qPCR SuperMix (Transgen, Beijing, China). The reaction conditions were set as follows: preincubation at 94 °C for 30 s, 40 cycles at 94 °C for 5 s, and 60 °C for 30 s, and melting at 60 °C for 60 s. The Cq values were evaluated using LightCycler^®^ 480 software (v1.5.1), and the relative expression patterns of target genes were calculated by the 2^−ΔΔCT^ method [38]. The *actin 4* gene was selected as the internal control for qRT-PCR detection [39]. The specific primers used in this study were designed by the Primer-BLAST tool (https://www.ncbi.nlm.nih.gov/tools/primer-blast/) (accessed on 1 August 2024) and are listed in Supplementary Table S6.

### 2.7. Determination of Plant Hormone Content

Hormone content analysis was undertaken by MetWare Biotechnology Co., Ltd. (Wuhan, China). using liquid chromatography–tandem mass spectrometry (LC-MS/MS). Samples comprising 0.1 g of freshly harvested leaf material from both the yellow and green regions of the same leaf were powdered and extracted with acetonitrile solution. A triple quadruple-tandem mass spectrometer (Quattro Ultima, Waters, USA) was used to detect the hormone levels. Analytical standards were sourced from Sigma (Missouri, USA), and the Metware Database (MWDB) was employed to facilitate the qualitative analysis of the mass spectrometry data. Each test was replicated three times.

### 2.8. Statistical Analysis

The data were expressed as the means ± SD from three independent biological replicates. A one-way ANOVA followed by a Duncan’s (D) test was performed on all data using SPSS software (v25.0), with a significance level of *p* < 0.05. Charts were generated via Origin Pro 2021 (v9.8.0) software.

## 3. Results

### 3.1. Comparison of Phenotype and Pigment Content

**Figure 1:**
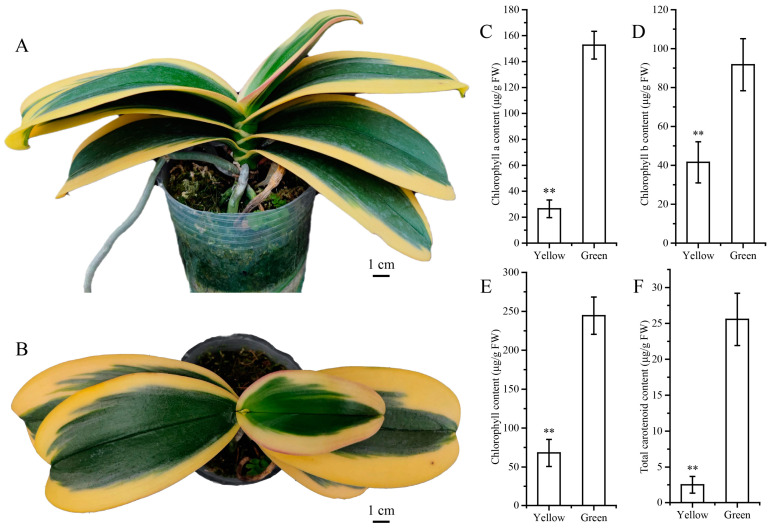
Phenotype characterization and pigment contents in the yellow and green regions of *Phalaenopsis* Chia E Yenlin Variegata leaves. (**A**,**B**) Phenotype of three-year *Phalaenopsis* Chia E Yenlin Variegata seedling. Chlorophyll a (**C**), chlorophyll b (**D**), total chlorophyll (**E**) and total carotenoid content (**F**) in the yellow and green region of leaves. Values are the mean ± standard deviation (n = 3). ** *p* < 0.01. Bar = 1 cm.

The leaves of *Phalaenopsis* Chia E Yenlin Variegata can be categorized into two regions based on the differences in leaf color: the yellow edge region and the central green region (Figure 1A). Interestingly, both young and mature leaves can maintain the bicolored characteristics for an extended period (Figure 1B). The yellowing process of green leaves is mainly influenced by the chlorophyll and carotenoid content. As a result, the content of chlorophyll a, chlorophyll b, total chlorophyll, and carotenoid in the GR was significantly higher than that of the YR. Compared to the GR, the total chlorophyll and carotenoid content in the YR exhibited a marked decrease of 72.18% and 90.21%, respectively, indicating that chlorophyll and carotenoid may play important roles in the formation of variegated leaves. (Figure 1C–F). The pigment ratio analysis between the YR and GR showed that the chlorophyll a-to-chlorophyll b ratios were 0.63 and 1.66, respectively, while the total-chlorophyll-to-carotenoids ratios were 27.17 in the YR and 9.56 in the GR.

### 3.2. Ultrastructure Observations

**Figure 2:**
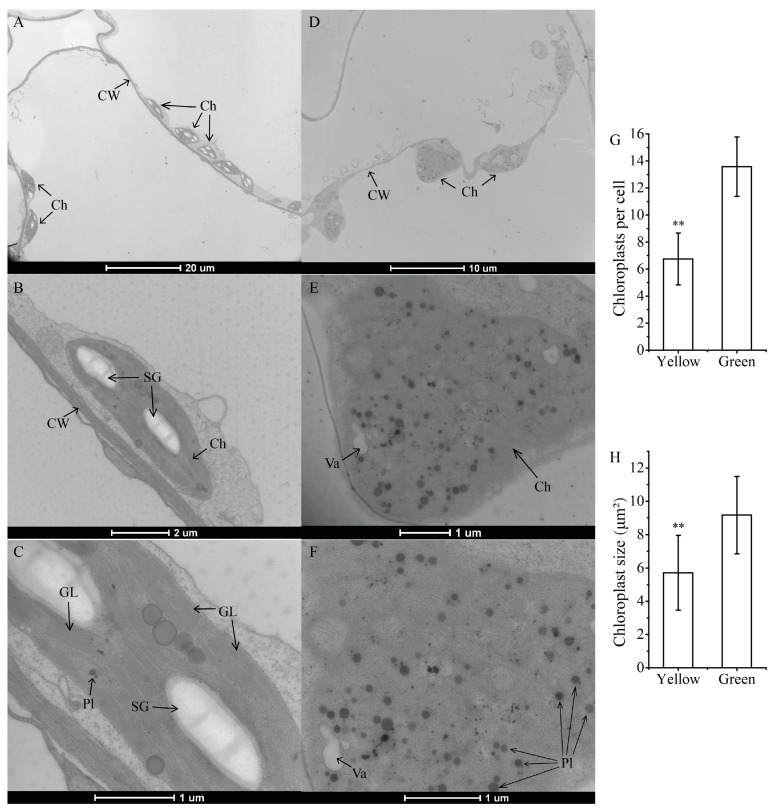
Transmission electron micrograph of chloroplast ultrastructure in different leaf-color regions. (**A**–**C**) Chloroplast ultrastructure structure of the mesophyll cells in the green region. (**D**–**F**) Chloroplast ultrastructure structure of the mesophyll cells in the yellow region. (**G**) Average number of chloroplasts per cell in the yellow and green regions. (**H**) Average chloroplast size in mutant leaves in the yellow and green regions. ** *p* < 0.01. Ch: chloroplast; CW: cell wall; GL: grana lamella; Pl: plastoglobuli; Va: vesicles; SG: starch granule.

We further examined the ultrastructure of chloroplasts in different leaf-color regions using transmission electron microscopy. In the GR, the chloroplasts exhibited normal and typical structures, dispersedly arranged and adjacent to the cell wall. The thylakoids and grana lamellae were clearly visible, with a greater number of stacked layers and a tight arrangement. Furthermore, the starch granules were relatively large and had a bright white elliptical shape (Figure 2A–C). In contrast, the plastid structure in the YR was disrupted, with no intact stroma lamellae or thylakoids, and the membrane structure was unclear and showed signs of degradation. There was a significant increase in irregularly arranged plastoglobuli, and a few vesicles were also observed (Figure 2D–F). Furthermore, the chloroplast number per cell and chloroplast size in the green region were significantly higher than those in the yellow-mutant region (Figure 2G,H). In conclusion, the ultrastructure of chloroplasts in the mutant leaves was destroyed, indicating that the development of chloroplasts was impaired, and the photosynthetic site was severely damaged.

### 3.3. Overview of RNA-Seq Data and Differentially Expressed Gene (DEG) Analysis

**Figure 3:**
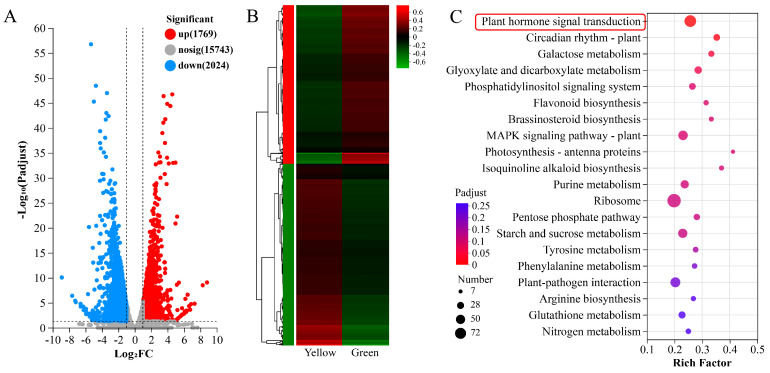
DEG analysis between different leaf color regions. (**A**) Volcano plots of the DEGs in the comparison group Yellow vs. Green. (**B**) Clustering heatmap of all DEGs from the transcriptome data. (**C**) KEGG analysis of the DEGs in the comparison group Yellow vs. Green. DEGs were screened using DESeq2 software and the red box indicates the most enriched KEGG pathway.

To elucidate the molecular mechanisms that contribute to the golden leaf coloration in *Phalaenopsis*, we performed transcriptome sequencing on a total of six samples of the YR and GR from the same leaf, using the Illumina platform. A total of 39.17 Gb of clean data was obtained after removing repetitive, low-quality, and adapter sequences. Both Q20 and Q30 values exceeded 97.93% and 94.26%, respectively, and the GC content ranged from 50.73% to 51.8% (Table S1). The total mapped ratio of each sample was more than 77.94% (Table S2) after the clean reads were compared with the *Phalaenopsis equestris* genome (Reference genome version: GCF_001263595.1). The gene expression correlation between each replicate showed a high coefficient (0.795–0.993) by principal component analysis (PCA) (Figure S1), providing evidence for the reliability of the sequence results. The transcriptome raw data have been uploaded in the National Center for Biotechnology Information (NCBI) database under accession number PRJNA1091187. A total of 23,206 expressed genes were detected, including 3670 new genes and 19,318 genes annotated in six public databases (GO, KEGG, COG, NR, Swiss-Prot, Pfam) (Figure S2). We screened for DEGs between the comparison of the yellow and green region (fold change ≥ 2, FDR < 0.05), and a total of 3793 DEGs were screened with 1769 upregulated and 2024 downregulated genes (Figure 3A,B). A KEGG enrichment analysis of DEGs revealed that the most significantly enriched pathway was ‘plant hormone signal transduction’ (54 genes, 25.7%), suggesting that plant hormones may play an important role in leaf yellowing.

### 3.4. Analysis of DEGs Involved in Chlorophyll and Carotenoid Biosynthesis

**Figure 4:**
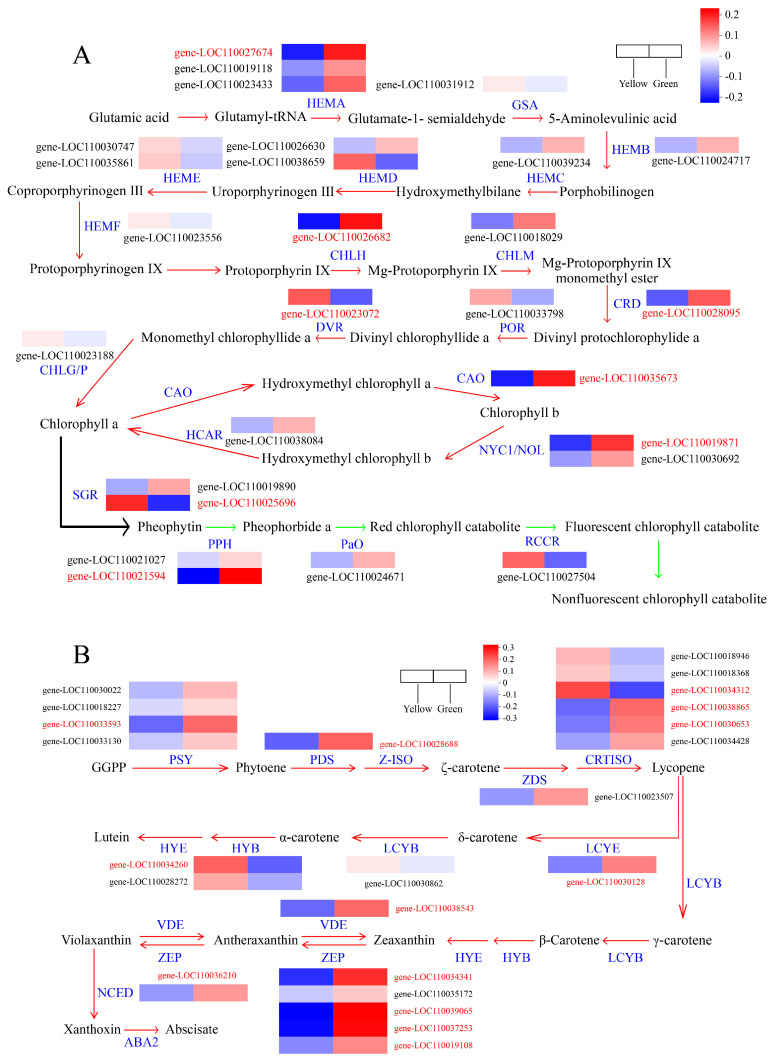
Expression profiles of genes involved in chlorophyll and carotenoid biosynthesis between different leaf-color regions. (**A**) Chlorophyll metabolism. The red and green arrows indicate the steps of Chlorophyll biosynthesis and degradation, respectively. (**B**) Carotenoid biosynthesis. DEGs were marked in red. The expression level was quantified from three biological replicates in the yellow and green regions, and the color bar indicates an increasing expression level from blue to red.

Serving as the primary constituent of pigmentation in plant leaves, the variances in gene expression pertinent to both the biosynthesis and degradation of chlorophyll can have a consequential impact on leaf color [40,41]. In this study, we identified 27 chlorophyll structural genes, of which 8 were identified as DEGs, and the gene expression patterns were displayed using a hierarchical cluster analysis (Figure 4A). One *HEMA* (gene-LOC110027674), which encodes glutamyl-tRNA reductase, was significantly downregulated in the YR, suggesting the obstruction of glutamate-1-semialdehyde biosynthesis in the YR. Furthermore, the expression of four chlorophyll biosynthesis DEGs, including *CHLH* (gene-LOC110026682), *CRD* (gene-LOC110028095), and *CAO* (gene-LOC110035673), in the GR was 2.01–2.68 times of that in the YR. Our findings suggested an association between the downregulated expression of the chlorophyll biosynthesis genes and reduced chlorophyll concentration in the YR. Meanwhile, a total of 13 DEGs were identified to participate in the carotene biosynthesis. Compared with the YR, the mRNA abundance of *PSY* (gene-LOC110033593), *PDS* (gene-LOC110028688), *CRTISOs* (gene-LOC110038865, gene-LOC110030653), *LCYE* (gene-LOC110030128), *ZEPs* (gene-LOC110034341, gene-LOC110039065, gene-LOC110037253, gene-LOC110019108), *VDE* (gene-LOC110038543), and *NCED* (gene-LOC110036210) manifested significant upregulation in the GR, whereas only two DEGs (*CRTISO* and *HYB*) were downregulated (Figure 4B). These findings implied that the gene expression levels of most carotenoid biosynthetic genes in the GR were markedly higher than those of the YR, which was consistent with the carotenoid content in green regions being much higher than in yellow.

### 3.5. DEG Analysis of Photosynthesis- and Carbon-Fixation-Related Genes

**Figure 5:**
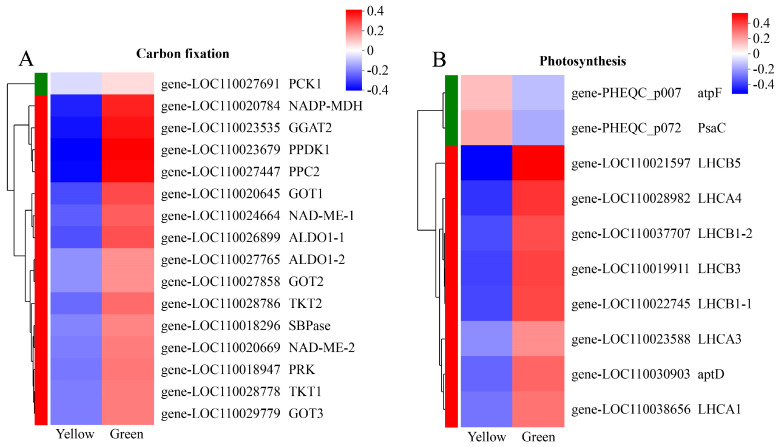
DEGs involved in carbon fixation and photosynthesis. (**A**) Carbon-fixation-related DEGs. (**B**) Photosynthesis-related DEGs. The expression level was quantified from three biological replicates in the yellow and green regions, and the color bar indicates an increasing expression level from blue to red.

To investigate the impact of leaf yellowing on carbon fixation and photosynthesis, the DEGs of related pathways were analyzed in more detail. In the carbon fixation in photosynthetic organisms (map00710) pathway, we identified 16 DEGs, all of which were downregulated in the YR of the leaves. Among them, the expression levels of PPC2 (phosphoenolpyruvate carboxylase 2-like), PPDK1 (pyruvate, phosphate dikinase 1), and GGAT2 (glutamate-glyoxylate aminotransferase 2-like) in the green parts were more than five times higher than in the yellow (Figure 5A). Furthermore, 10 DEGs were screened from 90 photosynthesis-related genes (map00195 and map00196). The mRNA abundance of atpF (ATP synthase CF0 B subunit) and PsaC (photosystem I subunit VII) was higher in the YR, while the remaining eight DEGs showed significant downregulation in the yellow sections compared to the normal green leaves, with downregulation ratios ranging from 3.01 to 11.32 times (Figure 5B). These results imply that biological carbon fixation and photosynthesis in the yellow sections of the leaves may be impaired.

### 3.6. DEG Analysis of Plant-Hormone-Related Genes

**Figure 6:**
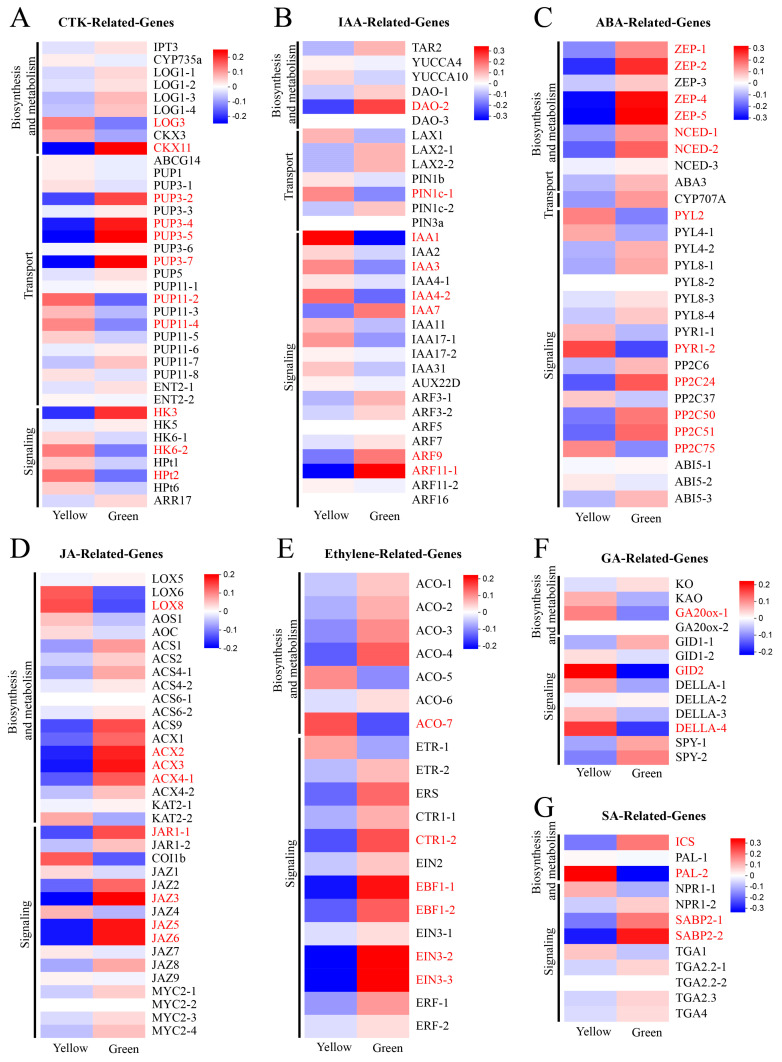
DEGs involved in plant hormone signaling pathways. The red text indicates the differentially expressed genes. (**A**) Cytokinin (CTK) (**B**) Auxin (AUX). (**C**) Abscisic acid (ABA). (**D**) Jasmonic acid (JA). (**E**) Ethylene. (**F**) Gibberellins (GA). (**G**) Salicylic acid (SA).

**Figure 7:**
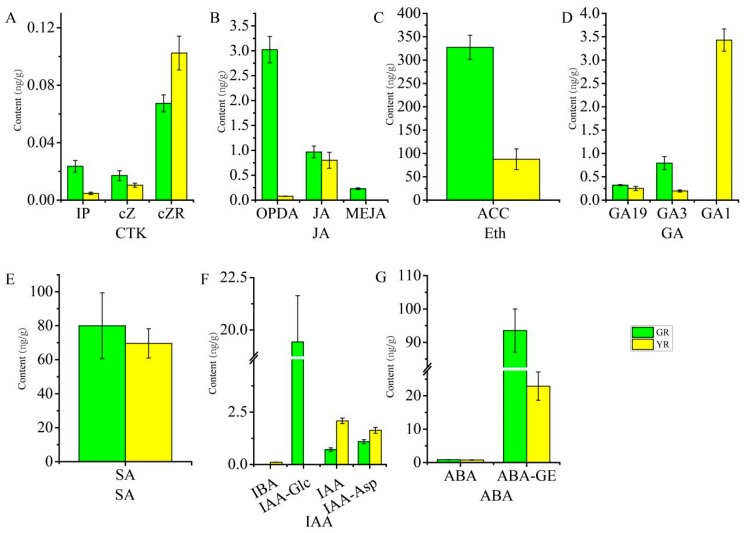
Determination of plant hormone content in the GR and YR. (**A**) CTK content. (**B**) JA content. (**C**) Eth content. (**D**) GA content. (**E**) SA content. (**F**) IAA content. (**G**) ABA content. The determination of hormone content was undertaken by the MetWare Biotechnology Co., Ltd. using a liquid chromatography–tandem mass spectrometry (LC-MS/MS) system, and the Metware Database (MWDB) was constructed for the qualitative analysis of mass spectrometry data. Three biological replications were performed for each test. IP: N6-isopentenyladenine; cZ: cis-Zeatin; cZR: cis-Zeatin riboside; OPDA: cis(+)-12-Oxophytodienoic acid; JA: Jasmonic acid; MEJA: Methyl jasmonate; ACC: 1-Aminocyclopropanecarboxylic acid; SA: Salicylic acid; IBA: Indole-3-butyric acid; IAA-Glc: 1-O-indol-3-ylacetylglucose; IAA: Indole-3-acetic acid; IAA-Asp: Indole-3-acetyl-L-aspartic acid; ABA: Abscisic acid; ABA-GE: ABA-glucosyl ester. CTK: Cytokinin, Eth: Ethylene, GA: Gibberellins, GA19: Gibberellin 19, GA3: Gibberellin 3, GA1: Gibberellin 1, GR: the green region, YR: yellow region.

Plant hormones play a crucial role in plant growth, development, metabolism, and responses to biotic and abiotic stresses [42,43,44]. To elucidate the expression patterns of plant-hormone-related genes in the green and yellow tissues of leaves, we identified a total of 202 hormone-related genes and 54 DEGs via comparison with the KEGG database. The cytokinin (CTK) and indole-3-acetic acid (IAA) pathways comprise 11 (5 upregulated, 6 downregulated) and 8 (4 upregulated, 4 downregulated) DEGs, respectively, with a predominant distribution in the transport and signaling components (Figure 6A,B). Twelve DEGs were identified in the abscisic acid (ABA) pathway, with the expression level of three genes upregulated and nine genes downregulated (Figure 6C). Eight DEGs were detected in jasmonic acid (JA)-related genes, of which only one lipoxygenase gene (LOX, gene-LOC110033226) was upregulated in the YR (Figure 6D). Similarly, we also identified only one 1-aminocyclopropane carboxylate oxidase (ACO, gene-LOC110035000) gene that was upregulated in the yellow parts among six ethylene-related DEGs (Figure 6E). A total of three DEGs (gene-LOC110031087, gene-LOC110026193, gene-LOC110034092) involved in the gibberellin (GA) pathway were identified, all of which were upregulated in the YR (Figure 6F). Additionally, one upregulated gene (gene-LOC110031047) and three downregulated genes (gene-LOC110033060, gene-LOC110024985, gene-LOC110025063) were isolated in the salicylic acid (SA) pathway of the YR (Figure 6G). Additionally, the actual concentrations of these hormones were consistent with the expression trends of the related genes (Figure 7).

### 3.7. TFs Involved in Leaf Yellowing

**Figure 8:**
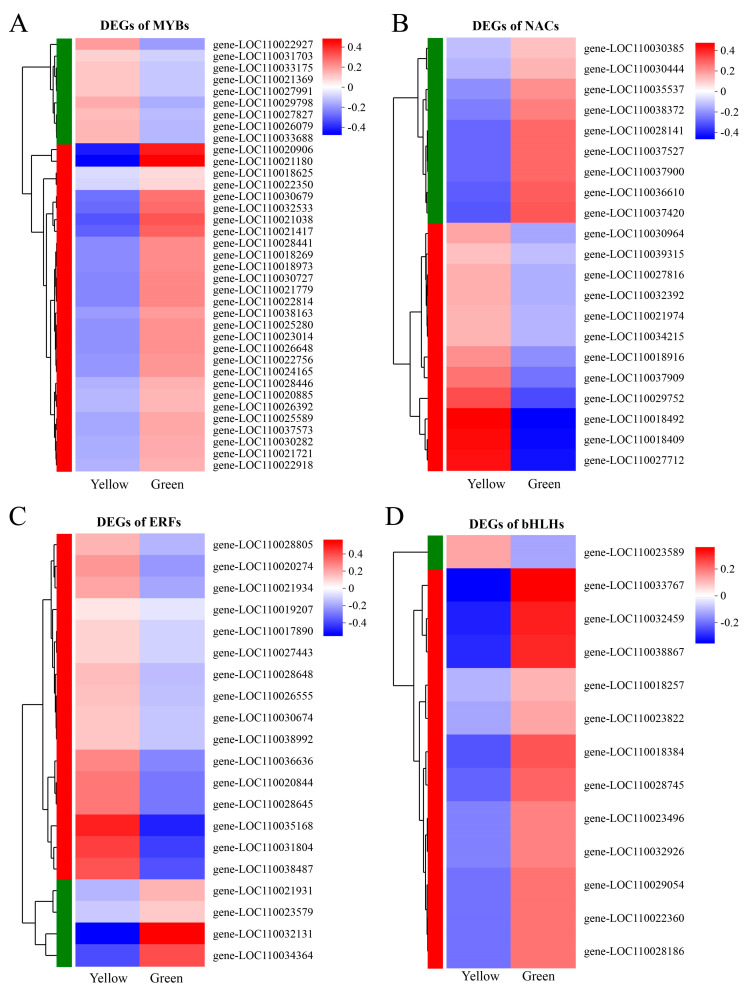
Heatmaps of differentially expressed transcription factors (TFs) in different leaf-color regions. (**A**) MYB TFs. (**B**) ERF TFs. (**C**) NAC TFs. (**D**) bHLH TFs.

Transcription factors (TFs) serve as crucial regulators by modulating the spatial and temporal expression of target genes through the activation or repression of their transcriptional activity, significantly influencing various plant biological processes. In this study, we annotated a total of 1201 TFs via comparison with the PlantTFDB database, of which 235 were identified as DEGs. We analyzed MYB, ERF, NAC, and bHLH TFs because they were reported to be involved in chlorophyll metabolism and leaf yellowing [14,15,17,45] (Figure 8). Among them, MYB TFs were the most highly represented DEG TFs, with a total of 37 annotated (9 upregulated and 28 downregulated) (Figure 8A). In total, 12 of the 21 DEGs in the NAC family were upregulated in the YR (Figure 8B). Additionally, the DEGs within the ERF and bHLH families exhibited contrasting expression profiles. Specifically, a majority of the DEGs in the ERF family showed upregulation (16 out of 20), whereas most genes in the bHLH family were downregulated (12 out of 13) in the YR. (Figure 8C,D).

### 3.8. Validation of RNA-Seq Data by qRT-PCR

**Figure 9:**
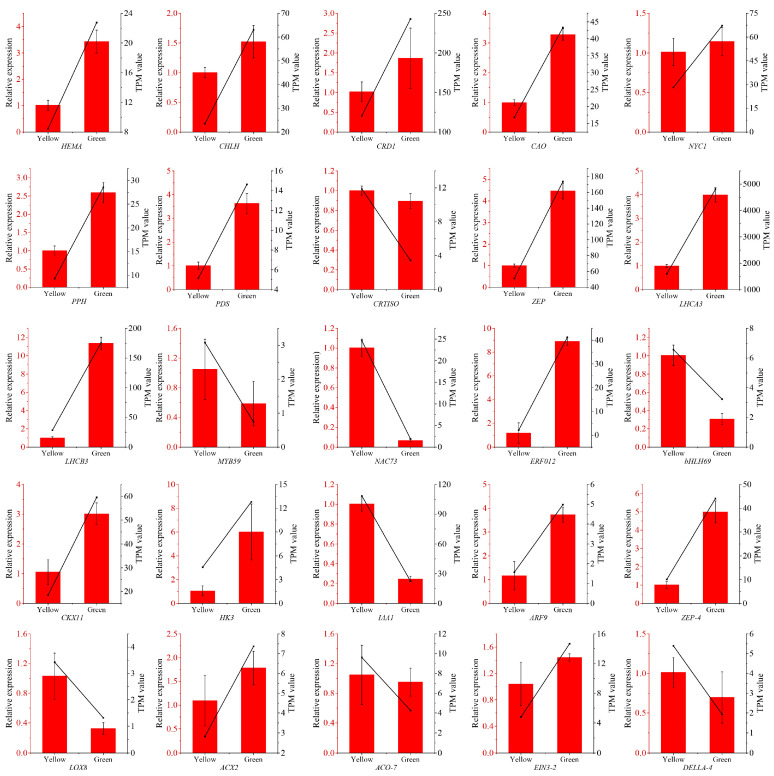
Real-time quantitative PCR (qRT-PCR) validation of the expression of 25 DEGs. The selected DEGs were involved in chlorophyll metabolism (*HEMA*, *CHLH*, *CRD1*, *CAO*, *NYC1*, *PPH*), carotenoid biosynthesis (*PDS*, *CRTISO*, *ZEP*), antenna proteins (*LHCA4*, *LHCB3*), TFs (*MYB59*, *NAC73*, *ERF012*, *bHLH93*, *bHLH69*), and plant hormones (*CKX11*, *HK3*, *IAA1*, *ARF9*, *ZEP-4*, *LOX8*, *ACX2*, *ACO-7*, *EIN3-2*, *DELLA-4*).

To further substantiate the accuracy of the RNA-Seq results, a qRT-PCR analysis was conducted on 25 DEGs, including five TFs (*MYB59*, *NAC73*, *ERF012*, *bHLH93*, *bHLH69*), nine structural genes associated with chlorophyll metabolism (*HEMA*, *CHLH*, *CRD1*, *CAO*, *NYC1*, *PPH*) and carotenoid biosynthesis (*PDS*, *CRTISO*, *ZEP*), two genes related to antenna proteins (*LHCA4*, *LHCB3*), and 10 genes involved in plant hormone pathways (*CKX11*, *HK3*, *IAA1*, *ARF9*, *ZEP-4*, *LOX8*, *ACX2*, *ACO-7*, *EIN3-2*, *DELLA-4*) (Figure 9). We calculated a significant correlation between the relative expression levels from the RNA-Seq and the qRT-PCR datasets, finding their correlation coefficient to reach 0.828 (Figure S3), indicating the high consistency of transcriptome data with qRT-PCR gene expression levels and suggesting that the RNA-seq results of the present study can be used for various downstream analyses.

## 4. Discussion

Leaf coloration is determined by the interaction of various pigments, with changes in the types and concentrations of these pigments determining the exhibited leaf color. Chlorophyll and carotenoids, for instance, confer green and yellow hues to leaves, respectively [46,47]. Previous research has demonstrated that chlorophyll levels in the leaves of yellowing mutants are significantly reduced compared to those in wild-type leaves across a diverse range of plant species [7,48,49]. In our study, we investigated a novel leaf variegation of *Phalaenopsis* named Chia E Yenlin Variegata, characterized by a yellow phenotype at the leaf margins. Quantitative analysis revealed that the chlorophyll content in the central green area was approximately 3.6-times higher than in the yellow peripheral area, suggesting that a reduction in chlorophyll content contributes to the observed changes in leaf color at the physiological level. In addition, the content of carotenoids in the GR was 10.22-times higher than that in the YR, similar to the pigment distributions observed in the yellowing mutants of other orchid species such as *Cymbidium* [50] and *Paphiopedilum* [9,51].

Chloroplasts are factories for photosynthesis and pigment biosynthesis, in which the thylakoid membranes are arranged neatly and orderly into grana. The structural integrity of the thylakoid membranes is crucial for the function of chloroplasts [52,53]. Studies have shown that the formation of leaf-color mutants may be related to abnormal chloroplast development. For instance, the chloroplast ultrastructure in mutant *Ilex* × *altaclerensis* leaves exhibited significant alterations, characterized by a reduced number of thylakoid lamellae and a disordered arrangement [54]. In *Ginkgo biloba*, ultrastructural analysis of chloroplasts in the yellow-mutant leaves demonstrated ruptured thylakoid membranes, indistinct or absent stromal lamellae, and chloroplasts densely packed with a multitude of vesicles and abundant plastoglobuli [49]. In *Cymbidium longibracteatum*, the cytological findings indicated that the plastid structure in the yellow-leaf varieties was severely damaged, with osmiophilic droplets aggregating and a complete absence of starch grains [55]. In this research, substantial differences were noted in the chloroplast ultrastructure between the YR and GR. YR chloroplasts were notably disrupted, characterized by the absence of intact stroma lamellae and thylakoids, along with an indistinct membrane structure exhibiting signs of degradation. Furthermore, there was a substantial increase in irregularly arranged plastoglobuli. Similar results were also found in the mutants of *Lagerstroemia indica* [55], *Arabidopsis* [56], and *Pseudosasa japonica* [57]. In summary, the chloroplast dysplasia in the YR contributes to the leaf-color variegation at the cytological level.

Comparative transcriptomic analysis can uncover changes in gene expression, revealing key genes and pathways involved in various biological processes [54,58]. RNA-Seq has also been applied to elucidate the mechanisms of leaf variegation in *Pelargonium zonale* [59], *Rosa beggeriana* [60], and *Ginkgo biloba* [49,61]. In the present study, we generated 39.17 Gb of clean data in six samples using an Illumina HiSeq 6000 sequencing platform. The proportion of filtered Q30 exceeded 93%, and the average GC content was 51.46%, aligning closely with a recent *Phalaenopsis* transcriptome study by Li et al. [62]. Furthermore, a total of 3793 DEGs were identified through mapping to the *Phalaenopsis equestris* reference genome, with 1769 genes upregulated and 2024 genes downregulated. The number of DEGs in this study surpasses that observed in the yellow-green-leaf mutant of maize (1122) [4] and yellow-leaf mutant of *Cymbidium* (1139) [8] but is less than the 4902 in the yellow-leaf tea plant [48].

Chlorophyll biosynthesis from glutamate-tRNA to chlorophyll a and b is facilitated by 15 enzymes, encoded by 27 genes. Alterations in any gene activity during chlorophyll biosynthesis and degradation can impact the efficiency of these biochemical processes, subsequently altering leaf coloration [63,64]. In *Arabidopsis*, when *HEMA1* mRNA abundance is reduced, the transgenic plants exhibit varying degrees of chlorophyll deficiencies, with leaf color ranging from patchy yellow to complete yellow [65]. Rice plants with mutations in *OsChlH* and *OsCRD1* display phenotypes of chlorina and chloroplast dysplasia [66,67]. The leaf color of maize and rice *cao1* mutants was yellow-green and pale green, respectively [40,68]. In addition, the *STAY-GREEN* (*SGR*) gene plays a crucial role in chlorophyll degradation. The *Arabidopsis sgr1* mutant and overexpressing plant exhibit a stay-green and yellowing phenotype, respectively [2,69]. The stay-green trait has been documented in *Capsicum annuum*, pea, rice, and *Medicago truncatula* [70,71,72,73]. Here, we identified six DEGs involved in chlorophyll metabolism, five of which (*HEMA*, *CHLH*, *CRD*, *CAO*, *NYC1/NOL*) were downregulated by 50.3–62.8% in the YR. Additionally, the expression of a *Stay-Green* gene (gene-LOC110021594), which is related to the promotion of chlorophyll degradation, was 2.37-times higher in the YR than that in the GR. Therefore, it could be considered that the concurrent downregulation of *HEMA*, *CHLH*, *CRD*, and *CAO* and the upregulation of the *SGR* gene decreases chlorophyll biosynthesis and accelerates its degradation, collectively reducing chlorophyll content and affecting leaf coloration.

Plant hormones play an important role in regulating chlorophyll metabolism and chloroplast development, thereby affecting leaf coloration [18,74,75,76]. CTK enhances chlorophyll biosynthesis, activates chloroplast development, and protects the photosynthetic apparatus [20]. When compared to wild-type *Arabidopsis* plants, the *ahk2 ahk3* cytokinin receptor mutants exhibited a decreased expression of chlorophyll biosynthesis genes, including *HEMA1*, *CHLH*, *GSA1*, *GUN4*, and *CHLM*, accompanied by reduced chlorophyll content [77]. In root tissues, cytokinin enhances chlorophyll level through the upregulation of the *GNC*, *CGA1*, and *GLK2* genes, a process that is dependent on the AHK2- and AHK3- receptors [78]. In the present work, six CTK-pathway-related DEGs (*CKX11*, three *PUP3*, and *HK3*) were significantly downregulated in the YR, suggesting a reduction in cytokinin levels that potentially influences leaf coloration. Furthermore, certain plant hormones, such as ethylene, jasmonic acid, salicylic acid, and abscisic acid, have been implicated in the induction of leaf senescence, which leads to leaf etiolation [79,80,81,82]. The rosette and cauline leaves of *EIN3ox* overexpression plants exhibited an accelerated yellowing phenotype and reduced longevity compared to wild-type plants [83]. Overexpression of *MdMYC2* significantly accelerated leaf senescence, with a more pronounced effect in the presence of exogenous MeJA, as evidenced by a further reduction in chlorophyll content [84]. However, the present data show that 83%, 75%, 87%, and 75% of the DEGs in the ethylene, salicylic acid, jasmonic acid, and abscisic acid pathways, respectively, were downregulated in the YR, and the concentrations of these hormones were also lower in the YR. Therefore, we hypothesize that the yellowing process at the leaf margins of *Phalaenopsis* may differ from the physiological process of leaf senescence or that these hormone-related genes may also be involved in other biological processes.

Considering that transcription factors (TFs) are proteins capable of binding to specific DNA sequences and regulating transcriptional cascades of numerous genes, it is vital to understand the impact of these TFs on leaf coloration in plants. Numerous studies have demonstrated that TFs are crucial regulators of chlorophyll metabolism [85,86,87]. Transgenic rice plants overexpressing OsMYB102 exhibited a stay-green phenotype during dark-induced leaf senescence with elevated chlorophyll content [17]. The stable overexpression of *Cymbidium sinense CsERF2* in *Nicotiana tabacum* resulted in reduced chlorophyll content and abnormal chloroplast development [15]. The banana NAC TF *MusaATAF2* induced leaf senescence by regulating chlorophyll degradation and H_2_O_2_ accumulation [14]. The bHLH TFs such as *PebHLH35* [45], *MdCIB1* [88], and *BvbHLH93* [89] were verified to enhance chlorophyll levels under abiotic stress conditions. In this study, a total of 235 differentially expressed TFs were identified, with 75.6% of MYB and 92.3% of bHLH TFs upregulated in the GR, while 80% of ERF TFs were upregulated in the YR. This provides a wealth of expression data for future research on the transcriptional regulation mechanisms in the leaf variegation of *Phalaenopsis*. Additionally, studies in *A*. *thaliana* [90], rice [91], tomato [92], and maize [93] have demonstrated that Golden2-Like (GLK) TFs acted as regulative factors for chloroplast development and the expression of photosynthetic genes. The expression of GLK TFs was downregulated in the yellow leaves of *G. biloba* and *Lagerstroemia indica* [49,55]. Similarly, we identified two GLK TFs from the transcriptome data, and their expression in the YR was decreased by 92.8% and 78.2% (Figure S4), respectively, compared to the GR, which suggests that the downregulation of GLK expression may inhibit the normal development of chloroplasts in the leaves, leading to structural differences compared to normal green leaves.

## 5. Conclusions

**Figure 10:**
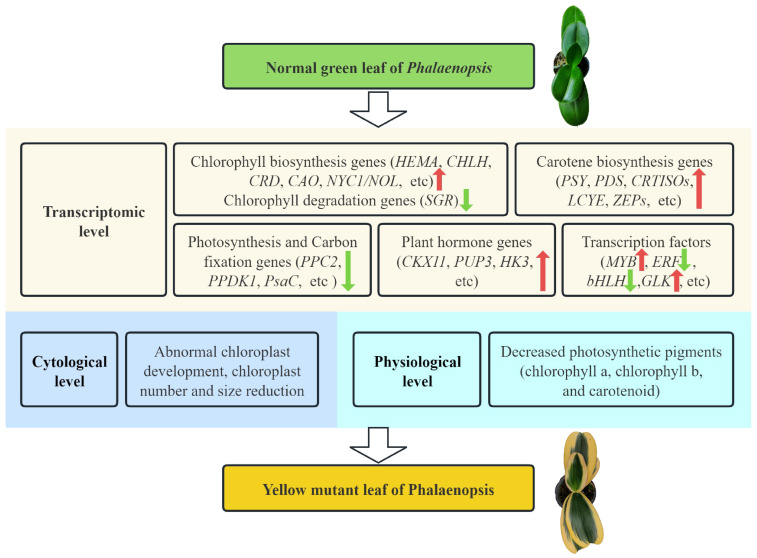
The proposed model elucidates the formation of leaf-color variegation in *Phalaenopsis* Chia E Yenlin Variegata.

In conclusion, this study explored the mechanisms behind the leaf-color variation in *Phalaenopsis* Chia E Yenlin Variegata through a comparative analysis between the green region (GR) and yellow region (YR) within the same plant at the cytological, physiological, and transcriptomic level. We observed a significant reduction in chlorophyll content and abnormalities in the chloroplast ultrastructure in the YR, suggesting a partial inhibition of chlorophyll biosynthesis and chloroplast development. Transcriptional sequence analysis identified a total of 3793 DEGs with 1769 upregulated and 2024 downregulated genes. Furthermore, the pathways related to chlorophyll and carotenoid metabolism, photosynthesis and carbon fixation, and plant hormone and transcription factors were analyzed, and changes in the expression of genes involved in these pathways might be responsible for the variegation in leaf color (Figure 10). Our findings offer valuable insights for further investigation into the functional roles of genes associated with leaf yellowing and for facilitating the breeding of new *Phalaenopsis* varieties with enhanced horticultural value.

## Data Availability

Data may be found within the article or Supplementary Materials. Raw reads were deposited in the NCBI database under BioProject number PRJNA1091187.

## Supplementary Materials

The following supporting information can be downloaded at https://www.mdpi.com/article/10.3390/biom14080963/s1, Table S1: Summary of sequencing data, Table S2: The results of RNA-Seq clean reads mapped to the reference sequence, Table S3: TPM values of chlorophyll- and carotenoid-metabolism-related genes, Table S4: TPM values of photosynthesis- and carbon-fixation-related genes, Table S5: TPM values of plant-hormone-related genes, Table S6: qRT-PCR primers used in the present study, Figure S1: Pearson correlation coefficient of the relative expression levels of all genes in each sample, Figure S2: Number of unigenes functionally annotated in six public databases, Figure S3: Correlation analysis based on RNA-seq data and real-time PCR, Figure S4: Expression profiles of GLK and SGR DEGs identified from the RNA-Seq database.

## Abbreviations

HEMA: glutamyl-tRNA reductase; GSA: glutamate-1-semialdehyde 2,1-aminomutase; HEMB: 5-aminolevulinate dehydrogenase; HEMC: porphobilinogen deaminase; HEMD: uroporphyrinogen III synthase; HEME: uroporphyrinogen III decarboxylase; HEMF: coproporphyrinogen III oxidase; HEMG: protoporphyrinogen oxidase; CHLH: Mg-chelatase; Mg-protoporphyrin IX methyltransferase; CHLM: Mg-protoporphyrin IX methyltransferase; CRD: Mg-protoporphyrin IX monomethylester cyclase; POR: protochlorophyllide oxidoreductase; DVR: divinyl chlorophyllide a 8-vinyl-reductase; CHLG: chl synthase; CAO: chlorophyllide a oxygenase; NYC1: chl b reductase; HCAR: hydroxymethyl Chl a reductase; SGR: STAY-GREEN (Mg-dechelatase); PPH: pheophytinase; PaO: pheophorbide a oxygenase; RCCR: red Chl catabolite reductase. GO: Gene Ontology; KEGG: Kyoto Encyclopedia of Genes and Genomes; COG: clusters of orthologous groups of proteins; NR: non-redundant protein sequence database; Swiss-Prot: a manually annotated, non-redundant protein sequence database; qRT-PCR: quantitative real-time polymerase chain reaction; PSY: phytoene synthase; PDS: phytoene desaturase; Z-ISO: zeta-carotene isomerase; ZDS: zeta-carotene desaturase; CRTISO: prolycopene isomerase; LYCE: lycopene epsilon-cyclase; LYCB: lycopene beta-cyclase; HYB: beta-ring hydroxylase; HYE: carotene epsilon-monooxygenase; VDE: violaxanthin de-epoxidase; ZEP: zeaxanthin epoxidase; NCED: 9-cis-epoxycarotenoid dioxygenase; ABA2: xanthoxin dehydrogenase.
