# Supplementary material for: Integrating Physiology, Cytology, and Transcriptome to Reveal the Leaf Variegation Mechanism in Phalaenopsis Chia E Yenlin Variegata Leaves
Source: Biomolecules. 2024 Aug 7;14(8):963. doi: 10.3390/biom14080963 (PMC11352648; doi:10.3390/biom14080963)

Supplementary Figure S1. Pearson correlation coefficient of relative expression levels of all genes in each sample.

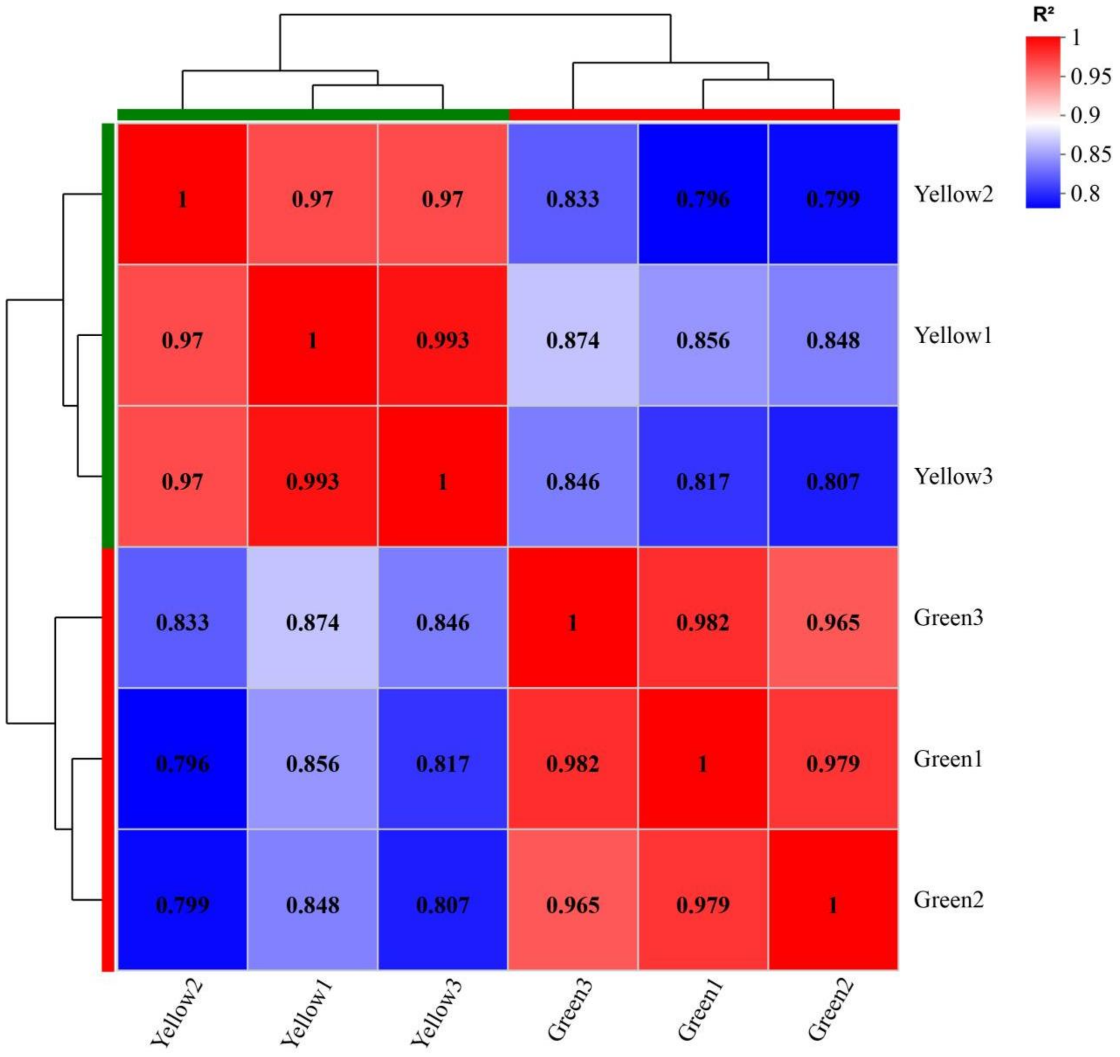

Supplementary Figure S2. Number of unigenes functionally annotated in six public databases.

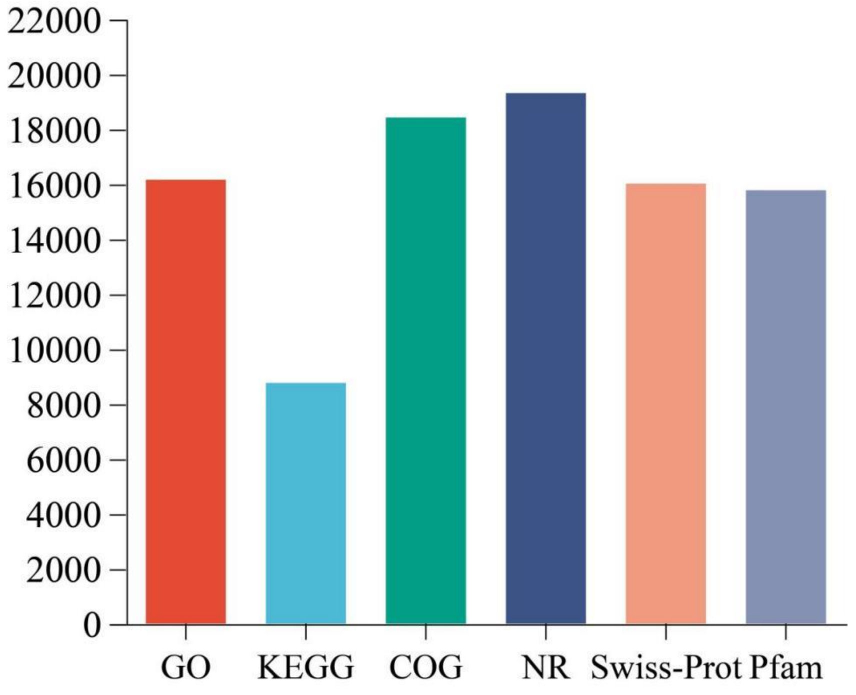

Supplementary Figure S3. Correlation analysis based on RNA-seq data and real-time PCR.

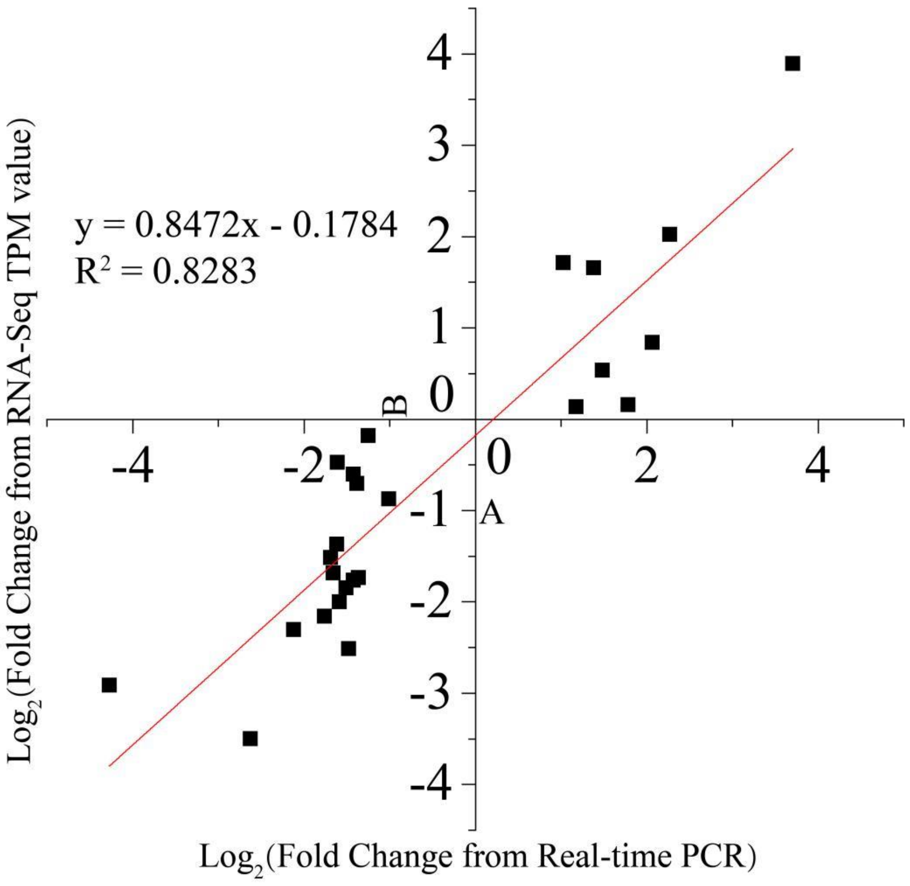

Supplementary Figure S4. Expression profiles of GLK and SGR DEGs identified from the RNA-Seq database.

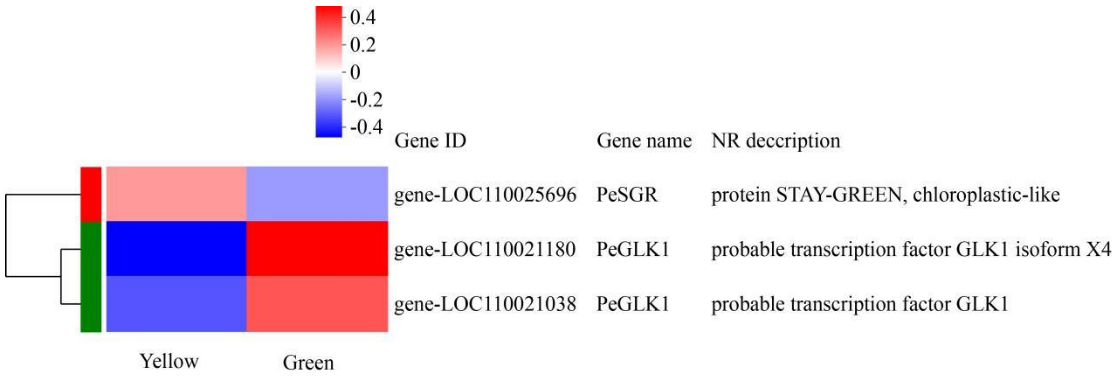

Supplement: Supplementary file 1 [file biomolecules-14-00963-s001.zip › Supplementary File-Figures.pdf]
